# Supplementary material for: Identification of Potential Targets Linked to the Cardiovascular/Alzheimer’s Axis through Bioinformatics Approaches
Source: Biomedicines. 2022 Feb 6;10(2):389. doi: 10.3390/biomedicines10020389 (PMC8962298; doi:10.3390/biomedicines10020389)
Supplement: Supplementary file 1 [file biomedicines-10-00389-s001.zip › biomedicines-1568638-supplementary.pdf]

Supplementary Materials

Supplementary Methods

Protein study for validation of the bioinformatic results

Protein extraction

Samples corresponding to 45 sections of femoral artery tissue were diluted in 500 μL of lysis buffer containing 20 mM Tris HCl pH 7.6, 10 mM NaCl, 1 mM EDTA, 4% SDS, 30% glycerol, 5 mM PMSF, 200 mM DTT and benzonase (1μL for each 10mL). The mixture was homogenized at 4°C by applying shear stress via a horn sonicator at 20% of amplitude and 7 cycles for 40s separated by a 20 s pause, in a Bandelin Sonoplus HD2070 sonicator and Bandelin UW 2070 horn tip. The obtained homogenate was centrifuged for 20 min at 4°C and 6000 rpm. The supernatant was decanted to a new vial and stored as cytosolic fraction at −80 °C.

Sample processing

Samples were prepared and analyzed in the Proteomics Facility at Research Support Central Service, University of Cordoba. Protein extracts were cleaned-up in 1D SDS-PAGE at 10% polyacrylamide. Previously, samples were concentrated by ultrafiltration using Amicon Ultra-0.5 Centrifugal Filter spin column with a cutoff of 3kDa. Then, samples were loaded onto the stacking gel and 100V was applied until the electrophoresis front reached the resolving gel. The run was stopped when the protein extract had entered 1 cm into the resolving gel and the gel was stained with Coomassie Blue. Proteins bands were cut off, diced and kept in water until digestion.

For protein digestion, gel dices were distained in 200 mM ammonium bicarbonate (AB)/50% acetonitrile for 15 min followed by 5 min in 100% Acetonitrile. Protein was reduced by the addition of 20 mM dithiothreitol in 25 mM AB and incubated for 20 min at 55ºC. The mixture was cooled down to room temperature, followed by alkylation of free thiols by addition of 40 mM iodoacetamide in 25 mM AB, in the dark for 20 min and the gel pieces were then washed twice in 25 mM AB. Proteolytic digestion was performed by the addition of 12.5 ng/uL Trypsin (Promega, Madison, WI, EE.UU.) in 25 mM AB and incubated at 37ºC overnight. Protein digestion was stopped by the addition of trifluoroacetic acid at 1% final concentration and the digested samples were finally Speedvac dried.

Nano-scale liquid chromatographic tandem mass spectrometry (nLC-MS/MS) Analysis

Nano-LC was performed in a Dionex Ultimate 3000 nano UPLC (Thermo Fisher Scientific) with a c18, 3um, 100A, 75 um i.d. x 50 Acclaim Pepmap100 column, nanoViper (Thermo Fisher Scientific). The peptide mix was previously loaded on a 300 um x 5mm Acclaim Pepmap precolumn (Thermo Fisher Scientific) in 2% acetonitrile/0.05% trifluoroacetic (TFA) for 5 min at 5uL/min. Peptide separation was performed at 40ªC for all runs. Mobile phase buffer A was composed of water, 0.1% formic acid. Mobile phase B was composed of 20% acetonitrile, 0.1% formic acid. Samples were separated at 300 nL/min. Elution conditions were: 4-35 % B for 120 min; 35-55 % B for 6 min; 55-90 % B for 3 min followed by 8 min wash at 90% B and a 15 min re-equilibration at 4% B. Total time of chromatography was 150 min.

Eluting peptide cations were converted to gas-phase ions by nano electrospray ionization and analyzed on a Thermo Orbitrap Fusion (Q-OT-qIT, Thermo Fisher Scientific) mass spectrometer operated in positive mode. Survey scans of peptide precursors from 400 to 1500 m/z were performed at 120.000 FWHM resolutions with 4 $x10^5$ ion count target. Tandem mass spectrometry was performed by isolation at 1.2 Da with the quadrupole, CID fragmentation

with normalized collision energy of 35, and rapid scan mass spectrometry analysis in the ion trap. The Automatic gain control ion count target was set to 2 x 10^3 and the max injection time was 300 ms. Only those precursors with charge state 2-5 were sampled for MS/MS. The dynamic exclusion duration was set to 15 s with a 10 ppm tolerance around the selected precursor and its isotopes. Monoisotopic precursor selection was turned on. The instrument was run in top 30 mode with 3 s cycles, meaning the instrument would continuously perform MS/MS events until a maximum of top 30 non-excluded precursors or 3s, whichever is shorter.

Protein Identification

The raw data were processed using Proteome Discoverer (version 2.1.0.81, Thermo Fisher Scientific). MS/MS spectra were searched with SEQUESTTM6 engine (Thermo Fisher Scientific) against Uniprot *Homo sapiens* database (www.uniprot.org). Peptides were generated by theoretical tryptic digestion allowing up to one missed cleavage, carbamidomethylation of cysteines as fixed modification and oxidation of methionine as post-translational variable modification. Precursor mass tolerance was 10 ppm and product ion were searched at 0.1 Da tolerance. Peptide spectral matches were validated using percolator based on q-values at 1% False discovery rate (FDR). With proteome Discoverer, the filtering threshold was 1% FDR for protein identification and minimal 1 peptide and 95% probability for peptide identification.

Unrecognized identifiers were manually curated using UniProtKB query main box. Any dubious association was dismissed and not further considered in downstream bioinformatics analysis.

Supplementary Table S1. DEGs identified between normal artery vs atherosclerotic artery using GEO2R.

Gene symbol	Gene name	Log FC	*Adj. p*-value	*Sequence*
MMP9	matrix metallopeptidase 9	4,110	1,63E-10	TGGAGGTGGGCTGGGCCCTCTCTTCTCACCTTTGTTTTTTGTTGGAGTGTT TCTAATAAA
CCL18	chemokine ligand 18	4,110	2,20E-09	ACATTCAATGCATGGATCAATCAGTGTGATTAGCTTTCTCAGCAGACATT GTGCCATATG
ACP5	acid phosphatase 5, tartrate resistant	3,900	1,02E-10	TTTGCTGAGTTCCGGGGTGCAATGGGGGAGGGAGGGAGGGAAAGCTTC CTCCTAAATCAA
-	-	3,260	9,07E-07	AGCAGACTACGAGAAACACAAAGTCTACGCCTGCGAAGTCACCCATCAG GGCCTGAGCTC
-	-	3,110	4,36E-07	AAACCCACCCATGTCAATGTGTCTGTTGTCATGGCGGAGGTGGACGGCA CCTGCTACTGA
-	-	3,090	4,61E-06	CATGAGGCTCTGCACAACCACTACACACAGAAGAGCCTCTCCCTGTCTCC GGGTAAATGA
IGLL5	immunoglobulin lambda-like polypeptide 5	3,040	2,18E-06	TAGTGTGTCTGATCAGTGACTTCTACCCGGGAGCTGTGACAGTGGCCTGG AAGGCAGATG
HBA2	hemoglobin, alpha 2	2,940	9,98E-08	TTCCTGGCTTCTGTGAGCACCGTGCTGACCTCCAAATACCGTTAAGCTGG AGCCTCGGTA
HBA2	hemoglobin, alpha 2	2,930	1,23E-07	GGCTTCTGTGAGCACCGTGCTGACCTCCAAATACCGTTAAGCTGGAGCCT CGGTAGCCGT
-	-	2,910	2,00E-06	AAAGCAACAACAAGTACGCGGCCAGCAGCTATCTGAGCCTGACGCCTGA GCAGTGGAAGT
-	-	2,880	2,21E-06	AGCCAACAAGGCCACACTGGTGTGCCTGATCAGTGACTTCTACCCGGGA GCTGTGAAAGT
HBA2	hemoglobin, alpha 2	2,870	2,93E-07	CACAGACTCAGAGAGAACCCACCATGGTGCTGTCTCCTGCCGACAAGAC CAACGTCAAGG
-	-	2,750	6,26E-06	AAGATAGCAGCCCCGTCAAGCGGGAGTGGAGACCACCACACCCTCCAA ACAAAGCAACAA
-	-	2,560	4,53E-06	ATACGAGTAGTGATCAACCGGTTTTCGGCGGAGGGACCAAGCTGACCGT CCTAGGTCAAG
IGLL1	immunoglobulin lambda-like polypeptide 1	2,530	2,61E-06	AACAAGGCCACACTGGTGTGTCTCATGAATGACTTCTATCTGGGAATCTT GACGGTGACC
HBD	hemoglobin, delta	2,520	2,56E-06	TGATGGCCTGGCTCACCTGGACAACCTCAAGGGCACTTTTTCTCAGCTGA GTGAGCTGCA

-	-	2,470	5,24E-06	CCATCAGCAGCCTGCAGTCTGAAGATTTTGCAGTTTATTACTGTCAGCAG TATAATAACT
CCL3	chemokine ligand 3	2,460	1,32E-07	TGCTTTTGTTCAGGGCTGTGATCGGCCTGGGGAAATAATAAAGATGCTCT TTTAAAAGGT
IBSP	integrin-binding sialoprotein	2,280	5,70E-06	TATACAGGGTTAGCTGCAATCCAGCTTCCCAAGAAGGCTGGGGATATAA CAAACAAAGCT
APOC1	apolipoprotein C-I	2,250	1,88E-04	GTTTTCAGAGACATTTCAGAAAGTGAAGGAGAAACTCAAGATTGACTCAT GAGGACCTGA
SPP1	secreted phosphoprotein 1	2,240	1,87E-05	TTCCACAGCCATGAATTTCACAGCCATGAAGATATGCTGGTTGTAGACCC CAAAAGTAAG
-	-	2,240	6,78E-05	TGAATGGCAAGGAGTACAAGTGCAAGGTCTCCAACAAAGCCCTCCCAGC CCCCATCGAGA
MMP12	matrix metallopeptidase 12	2,240	9,68E-05	TTGTCCATTCTTGCTTGACTCTACTATTAAGTTTGAAAATAGTTACCTTCAA AGGCCAAG
CCL4	chemokine ligand 4	2,220	1,06E-07	AAGTCTGTGCTGATCCCAGTGAATCCTGGGTCCAGGAGTACGTGTATGAC CTGGAACTGA
HMOX1	heme oxygenase 1	2,220	1,07E-07	TGGGGAGGGAGGTGTTTAACGGCACTGTGGCCTTGGTCTAACTTTTGTGT GAAATAATAA
-	-	2,220	3,68E-05	ACATCCAGATGACCCAGTCTCCATCCTCCCTGTCTGCATCTGTAGGAGGC AGAGTCACCA
-	-	2,200	5,53E-06	AGCAGCCTGCAGCCTGAAGATTTTGCAGTTTATTACTGTCAGCAGGATTA TAACTTACCT
-	-	2,200	1,13E-05	GGCAGCGGATATGGAAGAGATTTCACTCTCACTGTCAGCAGCCTGCAGC CTGAAGATTTT
CD52	CD52 molecule	2,190	2,16E-08	TGCCAGACATCACCAGGTTGTAGAAGTTGACAGGCAGTGCCATGGGGGC AACAGCCAAAA
CCL4	chemokine ligand 4	2,140	4,91E-07	CAGGAAGTCTTCAGGGAAGGTCACCTGAGCCCGGATGCTTCTCCATGAG ACACATCTCCT
-	-	2,140	2,51E-05	GTGGCACATACTATGCAGACTCCGTGAAGGGCCGATTCACCATCTCCAG AGACAATGCCA
-	-	2,120	5,76E-05	GACAGAGTCACCATCACTTGTCGGGCGAGTCAGGGAATTAGCAATTATTT AGCCTGGTTT
-	-	2,120	9,51E-05	GGGATGAGGCCGACTATTACTGTCAGGTGTGGGATAGTAGTAGTGATCA TCCCACGGTGA
PLA2G7	phospholipase A2, group VII	2,100	5,49E-07	AAAGCATTTAGGACTTCATAAAGATTTTGATCAGTGGGACTGCTTGATTG AAGGAGATGA

-	-	2,100	3,65E-05	CTCTCCTGCAGGGCCAGTCAGAGTGTTAGCGGCATCTACTTAGCCTGGTACCAGCAGAAA
-	-	2,050	2,79E-05	CTCCAGGCTGAGGACGAGGCTGATTATTACTGCTGCTCATATGCAGGTAGTAGCACTTTC
HBB	hemoglobin, beta	2,030	8,14E-06	GTCCAACTACTAAACTGGGGGATATTATGAAGGGCCTTGAGCATCTGGATTCTGCCTAAT
CCL3L3	chemokine ligand 3-like 3	2,020	7,21E-08	TTCCACAGAATTTCATAGCTGACTACTTTGAGACGAGCAGCCAGTGCTCCAAGCCCAGTG
LILRB4	leukocyte immunoglobulin-like receptor, subfamily B, member 4	2,010	4,26E-08	AAATATTACACATCAAACCAATGACATGGGAAAATGGGAGCTTCTAATGAGGACAAACAA
**APOE**	**apolipoprotein E**	**2,010**	**2,11E-05**	**CGCCCCAGCCGTCCTCCTGGGGTGGACCCTAGTTTAATAAAGATTCACCAAGTTTCACGC**
NCF1	neutrophil cytosolic factor 1	2,000	2,31E-10	GCCGAGCGCCGACCTCATCCTGAACCGCTGCAGCGAGAGCACCAAGCGGAAGCTGGCGTC
SLAMF8	SLAM family member 8	2,000	2,11E-08	TGCACCAGGGCCTTGTTGAACAGATCCACACTGCTCTAATAAAGTTCCCATCCTTAATGA
-	-	2,000	1,50E-05	TCCGGTCCGAGGATGAGGCTGATTATTACTGTGCAGCATGGGATGACAGCCTGAGTGGTC
-	-	2,000	3,79E-05	CATCACTGGTCTCCAGGCTGAGGACGAGGCTGATTATTACTGCAGCTCATATACAAGCAG
ITGAX	integrin, alpha X	1,990	5,88E-09	TTCATCGTGGGGCTCTCAGTTCCGATTCCCCAGGCTGAATTGGGAGTGAGATGCCTGCAT
C16orf54	chromosome 16 open reading frame 54	1,970	2,55E-12	GTATCACTTCTGTAAATGGAAAACCAGTCTCATTTGCCATCAATAGAAGGTAAAACATGA
AMICA1	adhesion molecule, interacts with CXADR antigen 1	1,960	4,22E-11	CTCCTGTGGGCAGGGTTCTTAGTGGATGAGTTACTGGGAAGAATCAGAGATAAAAACCAA
MARCO	macrophage receptor with collagenous structure	1,960	1,32E-04	TGGCTGGATAATGTTCAGTGTCGGGGCACGGAGAGTACCCTGTGGAGCTGCACCAAGAAT
ADAM8	ADAM metallopeptidase domain 8	1,950	1,47E-06	TATGTGGGAGAGTCAGCTATCTTGTCTGGTTTTCTTGAGACCTCAGATGTGTGTTCAGCA
LOC96610	BMS1 homolog, ribosome assembly protein pseudogene	1,950	3,70E-05	ATTTATGACAATAATAAGCGACCCTCAGGGATTCCTGACCGATTCTCTGGCTCCAAGTCT
TBC1D10C	TBC1 domain family, member 10C	1,940	2,25E-10	GGAAGGGGTTGGCTGAGTCAAGGGACCCCAGAGGGCACCAGGAATAAAATCTTCTTGAAC
SLC37A2	solute carrier family 37, member 2	1,930	6,92E-08	TAGCTCTTGGCATCTCCATCTGAGCCTAAAGTTGCCCACTGGCACCAATAGATTCTGTTT

MMP7	matrix metallopeptidase 7	1,920	2,16E-04	TTGGGTATGGGACATTCCTCTGATCCTAATGCAGTGATGTATCCAACCTATGGAAATGGA
IFI30	interferon, gamma-inducible protein 30	1,910	1,32E-07	ATGAAGCCCAGATACACAAAATTCCACCCCTAGATCAAGAATCCTGCTCCACTAAGAATG
-	-	1,910	3,22E-05	GGTGGTTATAACTATGTCTCCTGGTACCAACAGCACCCAGGCAAAGCCCCCAAACTCATG
-	-	1,900	1,67E-04	GATTTTACACTGAAAATCAGCAGAGTGGAGGCTGAGGATGTTGGGGTTTATTACTGCATG
SLAMF7	SLAM family member 7	1,890	1,53E-10	GGAGACCTCCCTACCAAGTGATGAAAGTGTTGAAAAACTTAATAACAAATGCTTGTTGGG
CD2	CD2 molecule	1,890	2,27E-09	GAGTTTCTTATGTGCCCTGGTGGACACTTGCCCACCATCCTGTGAGTAAAAGTGAAATAA
-	-	1,890	4,08E-05	TGATCTATGCTGCATCCAGTTTGCAGTCGGGGGTCCCATCTCGGTTCAGTGGCAGTGGAT
MPEG1	macrophage expressed 1	1,860	1,70E-10	CTAACCACTTGTCTGCAAGTACTGACTTTCCTATGAATTCTTTGAAGATTATTGAGTCAG
TNFRSF25	tumor necrosis factor receptor superfamily, member 25	1,860	1,06E-07	CGAGAGGGGGTGAAGACATTTCTCAACTTCTCGGCCGGAGTTTGGCTGAGATCGCGGTAT
IL1RN	interleukin 1 receptor antagonist	1,860	2,32E-05	TGCAAAGTTCCCTACTTCCTGTGACTTCAGCTCTGTTTTACAATAAAATCTTGAAAATGC
NFAM1	NFAT activating protein with ITAM motif 1	1,840	1,91E-09	CATATGTGGGTGACAGAAGCATATGTTACAGTGAAACATTAAAACTACAGCAAAGTGAAA
-	-	1,830	5,29E-05	ATCTCTTGTTCTGGAAGCAGCTCCAACATCGGAAGTAATACTGTAAACTGGTACCAGCAG
LOC100653210	ig kappa chain V-III region VG-like	1,820	5,91E-05	TGGTACCAACAGAAACCTGGCCAGGCTCCCAGGCTCCTCATCTATGATGCATCCAACAGG
NCF1	neutrophil cytosolic factor 1	1,810	1,31E-09	AGACGACGTCACAGGCTACTTCCCGTCCATGTACCTGCAAAAGTCAGGGCAAGACGTGTC
CECR1	cat eye syndrome chromosome region, candidate 1	1,800	8,78E-08	TAATCGGATATTTATTTCTGTGTCTACAGTCTTGCCCTGCCAGACTGTATGCCCCATGTG
IL4I1	interleukin 4 induced 1	1,800	3,44E-06	AAGGCCAGTTATCTCTCCAAAACACGACCCACACGAGGACCTCGCATTAAAGTATTTTCG
PLCB2	phospholipase C, beta 2	1,780	1,42E-09	GACAAGGTCAGCATCATTTGCTCTCCTGAATTTATGAGGTTTATTTATTTTTCTCTTTCC
FOLR2	folate receptor 2	1,770	9,72E-10	CCTTAAGCATGCTTCTATTAGTCACCTAACCCTCTGTCACCCAGTCTGTTGCTGCTCCAT

SAP25	Sin3A-associated protein, 25kDa	1,770	2,08E-08	CTTCTCAGGGCAGAGTGGGCTGGTTGTGTTGACAATAAAACAGTGTTGGT TTGCAAAAAA
IL10RA	interleukin 10 receptor, alpha	1,760	1,56E-10	CTGACTTGTCTAATTCATAGGGATGTGAGGTTCTGCTGAGGAAATGGGTA TGAATGTGCC
CSF1R	colony stimulating factor 1 receptor	1,740	1,56E-10	ATCCTAACTAACAGTCACGCCGTGGGATGTCTCTGTCCACATTAAACTAA CAGCATTAAT
CHI3L1	chitinase 3-like 1	1,740	1,82E-03	TCCTTATCAAAGGACACCATTTTGGCAAGCTCTATCACCAAGGAGCCAAA CATCCTACAA
-	-	1,730	5,57E-08	ATCCTCTATGAGATCTTGCTAGGGAAGGCCACCTTGTATGCCGTGCTGGT CAGTGCCCTC
TRAF3IP3	TRAF3 interacting protein 3	1,720	2,46E-10	TTTGTGACAACTGCCTTGGGTGAAAATCAGAAGCAAGCAACTCAGCGAA AAACTCAGAAG
MYO1F	myosin IF	1,720	7,67E-10	ATTTCTGTGTGTGTCAAAGGGGACTAACAGCAGAATCTACCTCCCAACTG CCATGTGATT
CSF3R	colony stimulating factor 3 receptor	1,720	1,70E-06	TCCATCCAGCCCCACCCAATGGCCTTTTGTGCTTGTTTCCTATAACTTCAG TATTGTAAA
CCR1	chemokine receptor 1	1,710	1,66E-07	CTTTTCAAGTTGGGTGATATGTTGGTAGATTCTAATGGCTTTATTGCAGCG ATTAATAAC
ITGB2	integrin, beta 2	1,700	1,39E-08	GCCAATTTATTTACATTTAAACTTGTCAGGGTATAAAATGACATCCCATTA ATTATATTG
STAB1	stabilin 1	1,690	3,11E-09	GTGGTTAGCCGTATCATTGTGTGGGACATCATGGCCTTCAATGGCATCAT CCATGCTCTG
S1PR4	sphingosine-1-phosphate receptor 4	1,690	3,74E-09	AGGCTGCAAGGGGTGGACTGTGGGATGCATGCCCTGGCAACATTGAAGT TCGATCATGGT
SLCO2B1	solute carrier organic anion transporter family, member 2B1	1,690	4,62E-08	TGCTGGTTGGTATCTGTAAATGTTTAATAAATATCTGAGCATGTATCTATC AACGCCAAG
CD163L1	CD163 molecule-like 1	1,680	1,96E-10	AATCACTGAATGCCTCCTCAGGTCATTTAGCACTTATTTTATCCAGTATCTT TGGGCTCC
IL7R	interleukin 7 receptor	1,680	5,02E-07	CATCCTGCTTCTACCATGTGGATTTGGTCACAAGGTTTAAGGTGACCCAA TGATTCAGCT
EGR2	early growth response 2	1,670	7,24E-08	AAGAATGTACATAATGTTACCGGAGCTGATTTGTTTGGTCATTAGCTCTTA ATAGTTGTG
-	-	1,670	4,84E-05	TTCACCATCTCCAGAGACAATTCCAAGAACACGCTGTATCTGCAAATGAA CAGCCTGAGA
-	-	1,670	2,66E-04	CTGGGTTCGCCAGGCTCCAGGGAAGGGACTGGAGTGGGTTTCATACATT AGTGGTAATAG

-	-	1,660	7,35E-05	TTGCCTGCAGTCTGAAGATTTTGCAACTTATTACTGTCAACAGTATTATAG TTTCCCTCC
FCGR3A	Fc fragment of IgG, low affinity IIIa, receptor	1,650	2,22E-08	TGCAGGGACTGTAAAACCACCTTTTCTGCTTCAATATCTAATTCCTGTGTA GCTTTGTTC
LAIR1	leukocyte-associated immunoglobulin-like receptor 1	1,650	3,49E-08	GGGGCAGTTGCTAATTTAGTTCTAGGCAAACGTGGACACATTAAATTCTC CTACAAACCC
COL1A1	collagen, type I, alpha 1	1,650	1,86E-06	GGTGGGAGGAAGCAAAAGACTCTGTACCTATTTTGTATGTGTATAATAAT TTGAGATGTT
RASAL3	RAS protein activator like 3	1,640	1,50E-10	ACAGTGGGGAGTGGAGCTGCTGGTCCCAACCACTCTGGCAGTATGAAGT TGCCCAGTAAA
CD83	CD83 molecule	1,640	1,08E-05	GCAGAAGGGACTTCACGAAGTGTTGCATGGATGTTTTAGCCATTGTTGGC TTTCCCTTAT
ADAP2	ArfGAP with dual PH domains 2	1,630	8,57E-10	AATGGAGGCATTGCAATGAAAAGGCACCCACAGCATCATGCAAGTGGCA TCTTGTAAAAA
ITGB7	integrin, beta 7	1,630	1,09E-08	CACCCTACTTCATTTTCAGAGTGACACCCAAGAGGGCTGCTTCCCATGCC TGCAACCTTG
CCL3L3	chemokine ligand 3-like 3	1,630	2,44E-06	AAGAGTAGTCAGTCCCTTCTTGGCTCTGCTGACACTCGAGCCCACATTCC ATCACCTGCT
HMHA1	histocompatibility HA-1	1,620	1,53E-10	CCACCGTGTGGTTCTTTCACAGGCACGTTTATTTTGCTGAAATAAAAAGTT TTTAATCGG
LILRA6	leukocyte immunoglobulin-like receptor, subfamily A, member 6	1,620	7,35E-07	CCAGATTCATCTGTGTTGCTGAACATGGTACAATTTCCTTCTTTTTATGCCT GAATATTA
FBP1	fructose-1,6-bisphosphatase 1	1,620	7,43E-06	TAATGCCACTGGTGTTAAGATATATTTTGAGTGGATGGAGGAGAAATAAA CTTATTCCTC
LGALS2	lectin, galactoside-binding, soluble, 2	1,620	3,76E-05	CTGAGCTACCTGAGCGTAAGGGGCGGGTTCAACATGTCCTCTTTCAAGTT AAAAGAATAA
CCR5	chemokine receptor 5	1,610	4,22E-10	AACAGTAGCATAGGACCCTACCCTCTGGGCCAAGTCAAAGACATTCTGA CATCTTAGTAT
CCL5	chemokine ligand 5	1,600	2,41E-07	AGATGAGCTAGGATGGAGAGTCCTTGAACCTGAACTTACACAAATTTGCC TGTTTCTGCT
ANXA8L2	annexin A8-like 2	1,600	2,03E-05	GGCAACTGAGACTGGGTACCTGGAGATTCTGAAGTGCCTTTGCTGTGGTT TTCAAAATAA
IGJ	immunoglobulin J polypeptide, linker protein for immunoglobulin alpha and mu polypeptides	1,600	2,16E-04	TTGGGTGATGTAAAACCAACTCCCTGCCACCAAAATAATTAAAATAGTCA CATTGTTATC
HBG1	hemoglobin, gamma A	1,600	1,34E-03	ACTGAGCTCACTGCCCATGATGCAGAGCTTTCAAGGATAGGCTTTATTCT GCAAGCAATA

LAPTM5	lysosomal protein transmembrane 5	1,590	3,94E-07	CATCATAAAACAGTCCCTTCAAACACACAATTGTTCTGCTGAAGAGTTGT CATCAACAAT
CD36	CD36 molecule	1,580	3,98E-06	CTTTGGCTTAATGAGACTGGGACCATTGGTGATGAGAAGGCAAACATGTT CAGAAGTCAA
TRAF3IP3	TRAF3 interacting protein 3	1,570	1,67E-09	CTGTGAAAAAGGGTTTCTATTCTCTCTGAAAGCACATGTCTGTGTTGAACA TTTCAATAA
LTB	lymphotoxin beta	1,570	1,67E-09	GGAAATTGATTTTGAACCTGATGAAAATAAAGAATGGAAAGCTTCAGTGC TGCCGATAAA
FGD3	FYVE, RhoGEF and PH domain containing 3	1,570	2,33E-09	CCTAGCTGGACTCATGGTTCCTAAATAACCACGCTCAGAAGCTCTGCTAG GACTTACCCC
CD68	CD68 molecule	1,570	1,18E-06	GGGTACCCTTATTTCCTCGACACGCAACTGGCTCAAAGACAATGTTATTTT CCTTCCCTT
HBQ1	hemoglobin, theta 1	1,570	1,61E-06	TGGAAAGGACCTTCCTGGCTTTCCCCGCCACGAAGACCTACTTCTCCCAC CTGGACCTGA
ARHGAP9	Rho GTPase activating protein 9	1,560	9,30E-10	CCCTTTAAATCTCCCAAATGACTGTCTCTATCTTCATGAGTGTGACTTGAG GTGTTGGGA
SYK	spleen tyrosine kinase	1,560	3,19E-09	ATACAGGTTATTTTTACGATCTGTTTCCAAATCCCTTTCATGTCTTTCCACT TCTCTGGG
TYROBP	TYRO protein tyrosine kinase binding protein	1,560	4,01E-08	GAGTGCCATCCCTGAGAGACCAGACCGCTCCCCAATACTCTCCTAAAATA AACATGAAGC
HLA-DRB1	major histocompatibility complex, class II, DR beta 1	1,560	5,71E-08	TTCGTGCTGGGCCTGCTCTTCCTTGGGGCCGGGCTGTTCATCTACTTCAG GAATCAGAAA
APOBR	apolipoprotein B receptor	1,560	1,11E-07	GCAGGCAAAACCAGACGTCTGGGAATACCGTGAACTTAAGGAGTCTGAT TCTCCGACACA
-	-	1,560	2,51E-04	TATTAATAGTTGGTTGGCCTGGTATCAGCAGAAACCAGGGAGAGCCCCT AAACTCCTAAT
ARHGAP25	Rho GTPase activating protein 25	1,550	1,50E-10	CATCTCTGAGACACAGGGGCAGAAAATGACATTCATCTTTTGAGTCCTCA TCCATGGAGT
LSP1	lymphocyte-specific protein 1	1,550	1,16E-09	CCCAGATCCAAGGGGAAACTGCAGGTCAAGGGCTGATAACGGCCATGC AGGATGCTTGAT
C2	complement component 2	1,550	3,64E-08	TTCTACCTCTGAATGGCCACCCTTAGACCCTGTGATCCATCCTCTCTCCTA GCTGAGTAA
CD84	CD84 molecule	1,550	5,38E-07	ATTATGAACGGATACATGCCTTAGGTCCGAACTACAATCTGGTCATTAGC GATCTGAGGA
AQP9	aquaporin 9	1,550	1,00E-04	TCTACATCAAGGGATGCACCTTCAGTCAAACTGTCAAAAAGCCCAGAATT CCCAAAGGCA

HLA-DMB	major histocompatibility complex, class II, DM beta	1,540	1,30E-07	GGACTATGCTGTAACCAAATTATTGTCCAAGGCTATATTTCTGGGATGAATATAATCTGA
ARHGAP27	Rho GTPase activating protein 27	1,530	2,39E-11	CACAGGGGCCTCATACCATATGTCTAAATATTTAAAAGTTATCAATCAAGCTAACAACTG
FAM78A	family with sequence similarity 78, member A	1,530	1,67E-09	AACCAATGCACTTACCAGGAGGCACAAGACTCTTGAAGAAGATGTAAAATGAACCTTTTT
PTAFR	platelet-activating factor receptor	1,530	2,15E-08	ATACGGTCACTGAAGTGGTTGTGCCATTCAACCAGATCCCTGGCAATTCCCTCAAAAATT
RNASE1	ribonuclease, RNase A family, 1	1,530	2,16E-07	GTTAGGGCTCCTATTCAACACACACATGCTTCCCTTTCCTGAGTCCCATCCCTGCGTGAT
CYTH4	cytohesin 4	1,520	1,15E-09	ACCTTGTGGGACATCTGAGGACATCCGCAGATTCTTGTCAGCCTGTGAACTAGGCCCTGC
CEBPA	CCAAT/enhancer binding protein, alpha	1,520	7,21E-08	GCCTTGTTTGTACTGTATGCCTTCAGCATTGCCTAGGAACACGAAGCACGATCAGTCCAT
CTSB	cathepsin B	1,520	3,82E-07	TCCTTCCAGTTTCAAGTAGAATATATTCATAACCTCAATAAAGTTCTCCCTGCTCCCAAA
TREM1	triggering receptor expressed on myeloid cells 1	1,520	7,31E-06	AACCTTACAAATGTGACAGATATCATCAGGGTTCCGGTGTTCAACATTGTCATTCTCCTG
HLA-DPB1	major histocompatibility complex, class II, DP beta 1	1,520	8,14E-06	CATTTGCTGTGTTTCGTTAGCATCTGGCTCCAGGACAGACCTTCAACTTCCAAATTGGAT
-	-	1,520	9,42E-05	AGGCCCCTGGACAAGGGCTTGAGTGGATGGGATGGATCAGCGCTTACAATGGTAACACAA
APBB1IP	amyloid beta precursor protein-binding, family B, member 1 interacting protein	1,510	3,70E-07	GTGATGGGAAACTTCTCACTGATGTGCTCAAGTACAGGCATAACCATTAACCCAGTAGAG
HLA-DQA1	major histocompatibility complex, class II, DQ alpha 1	1,510	3,81E-04	TGCGTTCAGTTGGTGCTTCCAGACACCAAGGGCCATTGTGAATCCCATCCTGGAAGGGAA
TMEM37	transmembrane protein 37	1,500	4,90E-09	AGCACGTCTGTACTTCTGTTCATTAAAGTGCTCCCTTTCTAGTCCTTTTTCTGCCCAGAA
SPI1	spleen focus forming virus proviral integration oncogene spi1	1,500	9,23E-09	ACCCGGGGTACTGCCTTGGGAGTCTCAAGTCCGTATGTAAATCAGATCTCCCCTCTCACC
LILRB3	leukocyte immunoglobulin-like receptor, subfamily B, member 3	1,500	2,34E-07	GCTGGGAACTTGTGGGACTCACCTGACTCAAAGATGACTAATATCGTCCCATTTTGGAAA
CD300A	CD300a molecule	1,500	5,58E-07	AGTTTCTCTGGACTCTTAGGTTTATTTTTAATATGAAATATAAAAACAGTTTCAAATATC
HS3ST2	heparan sulfate 3-O-sulfotransferase 2	1,500	1,31E-05	GATGATAGATATTATAAGCGATGATGGTTCTGTTGCTATGAACACAGCAGTCGGTCCCTG

CXCL1	chemokine ligand 1	1,500	3,99E-04	CATACTGCCTTGTTTAATGGTAGTTTTACAGTGTTTCTGGCTTAGAACAAAGGGGCTTAA
C13orf33	chromosome 13 open reading frame 33	-1,510	3,01E-06	ATTGGACACGGCAGCGTCCTCCTTATTGAAAACACATTATGTCAGTTGGGAATTTTAAAT
SERPINA3	serpin peptidase inhibitor, clade A, member 3	-1,520	7,44E-05	CATGGACTCTTCAGTCTGGAGGGTCCTGGGCCTCCTGACAGCAATAAATAATTTCGTTGG
ADAMTS4	ADAM metallopeptidase with thrombospondin type 1 motif, 4	-1,530	7,43E-03	AAATTGAATTCTACTATTTATGTGATCCTTTTGGAGTCAGACAGATGTGGTTGCATCCTA
SERPINA3	serpin peptidase inhibitor, clade A, member 3	-1,540	5,95E-05	ATAGGTGAGCTCTACCTGCCAAAGTTTTCCATCTCGAGGGACTATAACCTGAACGACATA
LINC00312	long intergenic non-protein coding RNA 312	-1,550	1,91E-06	AGTGATATTTGCCTTAGGTCAGATTCTACTAGTTTAAAGCAAAACGATCTCCTGGTCTGA
APOD	apolipoprotein D	-1,560	5,17E-04	TCACTAATGGAAAACGGAAAGATCAAAGTGTTAAACCAGGAGTTGAGAGCTGATGGAACT
HPR	haptoglobin-related protein	-1,600	5,93E-05	AAGCTTTGATAAGAGCTGTGCTGTGGCTGAGTATGGTGTGTATGTGAAGGTGACTTCCAT
CSF3	colony stimulating factor 3	-1,760	7,87E-03	GGGTCCCACGAATTTGCTGGGGAATCTCGTTTTTCTTCTTAAGACTTTTGGGACATGGTT
MYOC	myocilin, trabecular meshwork inducible glucocorticoid response	-1,850	3,75E-03	ATGCATTTACTACAGTTGGCTTCTAATGCTTCAGATAGAATACAGTTGGGTCTCACATAA
FAM150B	family with sequence similarity 150, member B	-1,860	3,47E-05	GACTCTTCCATAAGTCCTTTGAGTTTTGTATGTTGTTGACAGTTTGCAGATATATATTCG
HSPB7	heat shock 27kDa protein family, member 7	-1,900	2,86E-08	TATATAGATGGGGTTTTTCCAATACAGCTGGTTCGTGATAAACTGCATGAAACTCCTGCC
**HP**	**haptoglobin**	**-1,960**	**2,17E-04**	**GATAAGATGTGGTTTGAAGCTGATGGGTGCCAGCCCTGCATTGCTGAGTCAATCAATAAA**
SCARA5	scavenger receptor class A, member 5	-2,020	3,31E-03	CAATATGCTTGCCACTCCTTAAATGTCCTAATGATGAGAAACTCTCTTTCTGACCAATTG
PCP4	Purkinje cell protein 4	-2,090	3,40E-06	CCCTCCTAGTCCACCTGAAAACACCAAATTCAACCATCATCTGTCAAGAAATTAAAAGAA
SAA2	serum amyloid A2	-2,170	2,34E-05	CTATGTCCAGAGAAGCTGAGATATGGCATATAATAGGCATCTAATAAATGCTTAAGAGGT
PI16	peptidase inhibitor 16	-2,190	2,74E-04	AGGGACGAGGGAAGGAAAGTAACTCCTGACTCTCCAATAAAAACCTGTCCAACCTGTGGC
DES	desmin	-2,250	2,20E-04	CTCCCCTCCCCTGCTGCAGGGGCTCTGGAGAGAAACAATAAAGAGATTCACACACAAGCC

SAA1	serum amyloid A1	-2,310	5,47E-05	CAGAAGCGATCAGCGATGCCAGAGAGAATATCCAGAGATTCTTTGGCCATGGTGCGGAGG
CXCL14	chemokine ligand 14	-2,430	3,09E-03	ACGAAGAATAGGGTGAAAAACCTCAGAAGGGAAAACTCCAAACCAGTTGGGAGACTTGTG
PLA2G2A	phospholipase A2, group IIA	-2,830	2,06E-04	AAGAACTCTTACCATGAAGACCCTCCTACTGTTGGCAGTGATCATGATCTTTGGCCTACT

FC: Fold Change; ''- '': No matching in genebank.

Supplementary Table S2. Results from GO and KEGG pathway enrichment analysis for the module linked to cardiovascular and Alzheimer's disease.

Category	Term	Count	*p*-value
GOTERM_CC_DIRECT	GO:0034361~very-low-density lipoprotein particle	17	1,44E-22
GOTERM_CC_DIRECT	GO:0034364~high-density lipoprotein particle	17	2,21E-21
GOTERM_BP_DIRECT	GO:0042157~lipoprotein metabolic process	17	2,35E-16
GOTERM_BP_DIRECT	GO:0008203~cholesterol metabolic process	17	1,45E-10
GOTERM_BP_DIRECT	GO:0042632~cholesterol homeostasis	16	6,81E-10
GOTERM_CC_DIRECT	GO:0005576~extracellular region	36	3,06E-08
GOTERM_BP_DIRECT	GO:0043691~reverse cholesterol transport	12	4,00E-09
GOTERM_CC_DIRECT	GO:0042627~chylomicron	11	2,14E-07
GOTERM_BP_DIRECT	GO:0034375~high-density lipoprotein particle remodeling	11	1,45E-06
GOTERM_BP_DIRECT	GO:0006869~lipid transport	15	1,19E-05
GOTERM_BP_DIRECT	GO:0006898~receptor-mediated endocytosis	18	8,74E-06
GOTERM_CC_DIRECT	GO:0034362~low-density lipoprotein particle	10	1,72E-05
GOTERM_BP_DIRECT	GO:0070328~triglyceride homeostasis	11	2,49E-03
GOTERM_CC_DIRECT	GO:0034366~spherical high-density lipoprotein particle	8	1,07E-01
GOTERM_BP_DIRECT	GO:0033344~cholesterol efflux	10	3,57E-02
GOTERM_BP_DIRECT	GO:0001523~retinoid metabolic process	12	4,71E-01
GOTERM_CC_DIRECT	GO:0072562~blood microparticle	14	4,53E-01
GOTERM_BP_DIRECT	GO:0034372~very-low-density lipoprotein particle remodeling	7	5,79E+00
GOTERM_BP_DIRECT	GO:0033700~phospholipid efflux	8	7,99E+00
GOTERM_MF_DIRECT	GO:0008289~lipid binding	13	4,18E+01
GOTERM_BP_DIRECT	GO:0006629~lipid metabolic process	13	7,13E+01
GOTERM_CC_DIRECT	GO:0005615~extracellular space	25	9,37E-01
GOTERM_MF_DIRECT	GO:0005319~lipid transporter activity	8	1,72E+03
GOTERM_BP_DIRECT	GO:0051006~positive regulation of lipoprotein lipase activity	7	1,73E+03
GOTERM_MF_DIRECT	GO:0015485~cholesterol binding	9	5,62E+02
GOTERM_MF_DIRECT	GO:0060228~phosphatidylcholine-sterol O-acyltransferase activator activity	6	1,66E+04
GOTERM_MF_DIRECT	GO:0034185~apolipoprotein binding	7	2,37E+03
GOTERM_MF_DIRECT	GO:0017127~cholesterol transporter activity	7	3,94E+03
GOTERM_CC_DIRECT	GO:0071682~endocytic vesicle lumen	7	3,98E+02
GOTERM_MF_DIRECT	GO:0005543~phospholipid binding	10	6,64E+03
GOTERM_CC_DIRECT	GO:0070062~extracellular exosome	31	1,18E+05
KEGG_PATHWAY	hsa03320:PPAR signaling pathway	10	1,45E+00
GOTERM_BP_DIRECT	GO:0006641~triglyceride metabolic process	8	1,49E+05
GOTERM_BP_DIRECT	GO:0034380~high-density lipoprotein particle assembly	6	1,58E+05
GOTERM_BP_DIRECT	GO:0042158~lipoprotein biosynthetic process	6	3,55E+04
GOTERM_BP_DIRECT	GO:0010873~positive regulation of cholesterol esterification	6	3,55E+04
GOTERM_BP_DIRECT	GO:0045723~positive regulation of fatty acid biosynthetic process	6	7,08E+03
GOTERM_BP_DIRECT	GO:0034374~low-density lipoprotein particle remodeling	6	1,29E+06
GOTERM_BP_DIRECT	GO:0019433~triglyceride catabolic process	7	1,40E+06
GOTERM_BP_DIRECT	GO:0010875~positive regulation of cholesterol efflux	6	5,57E+05
GOTERM_MF_DIRECT	GO:0055102~lipase inhibitor activity	5	1,40E+07
GOTERM_BP_DIRECT	GO:0034384~high-density lipoprotein particle clearance	5	1,43E+07
GOTERM_BP_DIRECT	GO:0034382~chylomicron remnant clearance	5	1,43E+07
GOTERM_MF_DIRECT	GO:0016209~antioxidant activity	6	4,14E+06
GOTERM_MF_DIRECT	GO:0031210~phosphatidylcholine binding	6	8,92E+06
GOTERM_BP_DIRECT	GO:0046470~phosphatidylcholine metabolic process	5	1,98E+08
GOTERM_MF_DIRECT	GO:0008201~heparin binding	9	3,93E+08
GOTERM_MF_DIRECT	GO:0005041~low-density lipoprotein receptor activity	5	6,54E+07

GOTERM_CC_DIRECT	GO:0043235~receptor complex	8	9,12E+07
GOTERM_BP_DIRECT	GO:0098869~cellular oxidant detoxification	7	9,30E+07
GOTERM_BP_DIRECT	GO:0006953~acute-phase response	6	1,51E+08
GOTERM_BP_DIRECT	GO:0030301~cholesterol transport	5	1,69E+09
GOTERM_CC_DIRECT	GO:0005769~early endosome	9	3,48E+08
KEGG_PATHWAY	hsa05143:African trypanosomiasis	5	1,63E+11
GOTERM_BP_DIRECT	GO:0002576~platelet degranulation	6	1,96E+11
GOTERM_CC_DIRECT	GO:0005905~clathrin-coated pit	5	2,10E+11
GOTERM_BP_DIRECT	GO:0050728~negative regulation of inflammatory response	5	1,20E+12
GOTERM_MF_DIRECT	GO:0019899~enzyme binding	7	6,55E+10
GOTERM_BP_DIRECT	GO:0006897~endocytosis	5	1,03E-03
GOTERM_CC_DIRECT	GO:0005788~endoplasmic reticulum lumen	5	2,50E-03
GOTERM_CC_DIRECT	GO:0005783~endoplasmic reticulum	9	2,86E-03
GOTERM_CC_DIRECT	GO:0005794~Golgi apparatus	9	3,68E-03
GOTERM_CC_DIRECT	GO:0005768~endosome	5	4,41E-03
GOTERM_CC_DIRECT	GO:0009986~cell surface	7	5,19E-03
KEGG_PATHWAY	hsa05010:Alzheimer's disease	5	8,08E-03
GOTERM_BP_DIRECT	GO:0042493~response to drug	5	1,64E-02
GOTERM_CC_DIRECT	GO:0030425~dendrite	5	1,73E-02
GOTERM_MF_DIRECT	GO:0005509~calcium ion binding	7	2,63E-02
GOTERM_MF_DIRECT	GO:0042802~identical protein binding	7	3,17E-02
GOTERM_BP_DIRECT	GO:0045087~innate immune response	5	4,92E-02
GOTERM_MF_DIRECT	GO:0005515~protein binding	35	6,98E-02
GOTERM_CC_DIRECT	GO:0043231~intracellular membrane-bounded organelle	5	8,30E-02

Count represents the gene number of the secondary protein-protein interaction network involved in the corresponding term; *p*-value consists of the modified Fisher exact p-value for the enrichment performed.
